# The effect of daily UVA phototherapy for 2 weeks on clinic and 24-h blood pressure in individuals with mild hypertension

**DOI:** 10.1038/s41371-022-00729-2

**Published:** 2022-08-05

**Authors:** Richard B. Weller, Iain M. Macintyre, Vanessa Melville, Michael Farrugia, Martin Feelisch, David J. Webb

**Affiliations:** 1 Centre for Inflammation Research and Edinburgh Skin Network, University of Edinburgh, Edinburgh, UK; 2 Department of Nephrology, Royal Infirmary of Edinburgh, Edinburgh, UK; 3 University Clinical Research Centre, Western General Hospital, Edinburgh, UK; 4 Department of Dermatology, Royal Infirmary of Edinburgh, Edinburgh, UK; 5 Clinical & Experimental Sciences, Faculty of Medicine, University of Southampton, Southampton, UK; 6 Centre for Cardiovascular Science, University of Edinburgh, Edinburgh, UK

**Keywords:** Risk factors, Hypertension, Clinical trials

## Abstract

Latitude and season determine exposure to ultraviolet radiation and correlate with population blood pressure. Evidence for Vitamin D causing this relationship is inconsistent, and temperature changes are only partly responsible for BP variation. In healthy individuals, a single irradiation with 20 J/cm^2^ UVA mobilises NO from cutaneous stores to the circulation, causes arterial vasodilatation, and elicits a transient fall in BP. We, therefore, tested whether low-dose daily UVA phototherapy might be an effective treatment for mild hypertension. 13 patients with untreated high-normal or stage 1 hypertension (BP 130-159/85-99 mm Hg), confirmed by 24-h ambulatory blood pressure (ABP), were recruited. Using home phototherapy lamps they were either exposed to 5 J/cm^2^ full body UVA (320–410 nm) radiation each day for 14 days, or sham-irradiated with lamps filtered to exclude wavelengths <500 nm. After a washout period of 3 ± 1 week, the alternate irradiation was delivered. 24-h ABP was measured on day 0 before either irradiation sequence and on day 14. Clinic BP was recorded on day 0, and within 90 min of irradiation on day 14. There was no effect on 24-h ABP following UVA irradiation. Clinic BP shortly after irradiation fell with UVA (−8.0 ± 2.9/−3.8 ± 1.1 mm Hg *p* = 0.034/0.029) but not sham irradiation (1.1 ± 3.0/0.9 ± 1.5 mm Hg). Once daily low-dose UVA does not control mildly elevated BP although it produces a transient fall shortly after irradiation. More frequent exposure to UVA might be effective. Alternatively, UVB, which photo-releases more NO from skin, could be tried.

## Introduction

Hypertension is a major risk factor for myocardial infarction, stroke, cardiac failure and peripheral vascular disease and is now the leading cause of Disability Adjusted Life Years (DALYs) globally [1]. Robust and extensive epidemiological data suggest that lack of sunlight is a major and largely unaccounted for risk factor for much cardiovascular disease [2].

Both latitude and season correlate strongly with blood pressure (BP). Incident ultraviolet (UV) radiation from the sun rises in intensity during summer and is inversely associated with latitude. Systolic and diastolic BP are higher in winter than summer in higher latitude countries [3], and this seasonal variation is accompanied by a 23% rise (95% CI: 1.16–1.31) in fatal cardiovascular events in winter compared with summer [4]. Although temperature has previously been considered to account for this seasonal variation, we have shown in the largest study to correlate observed BP with temperature and irradiance, that temperature only accounts for around half of the observed relationship between UV and BP [5].

UV irradiation alters expression of some BP regulatory genes [6], and we have also demonstrated a mechanism whereby UVA exposure causes a photochemical reduction of cutaneous stores of nitrogen oxides to nitric oxide (NO) which enters the systemic circulation and causes arterial vasodilatation [7].

We hypothesised that UVA radiation itself may lower BP and conducted a trial with UVA phototherapy lamps used once daily as a potential treatment for mild hypertension.

## Methods

### Recruitment

Our study was approved by the South East Scotland Research Ethics Committee (16/SS/0120) and registered with ClinicalTrials.gov (NCT02621866). We recruited patients diagnosed by their primary care doctor with ESC/ESH defined high-normal or stage 1 hypertension [8] (Clinic BP 130–159/85–99 mm Hg) using the NHS Research Scotland Primary Care Network. The diagnosis was confirmed with 24-h ABP at the screening visit. We aimed to recruit 80 such patients to give us adequate power to detect a 2 mmHg fall in ABP. Patients had to be aged over 16 and have given informed consent. Those with red hair, Fitzpatrick type 1 skin (always burns, never tans), a history of skin cancer, or taking photosensitising or anti-hypertensive medication were excluded.

### Design and intervention

The study used a randomised sham-controlled crossover design. Active treatment was 5 J/cm^2^ of UVA delivered from Waldmann UV100 home phototherapy lamps fitted with 8 of the same Waldmann F85/100 W UVA (320–410 nm) bulbs used in our previous study [7]. For the sham treatment, the bulbs were covered with a UV filter, preventing transmission of wavelengths <500 nm (Epak Electronics, Chard, Somerset). Lamps were delivered to the subjects’ homes, where they irradiated themselves once daily for 14 days with either an active or a sham lamp. The irradiation period was around 10 min to each side of the body. After a washout period of 3 ± 1 week the lamp was exchanged, and irradiation was repeated identically with the alternative lamp. The irradiation sequence was randomised, and subjects and investigator blinded to treatment allocation.

### Measurements

Baseline characteristics including Fitzpatrick skin type, constitutive skin colour and full physical examination were performed at the screening clinic visit and instruction given on use of the home phototherapy lamps. Informed consent was taken. On day 1 and day 15 of the irradiation sequences, clinic BP and skin colour were recorded, and blood drawn for serum vitamin D (both 25(OH)D2 and 25(OH)D3), nitrite and nitrate levels. The day 1 measurements were taken before any irradiation. Day 15 measurements were taken after the final irradiation, when participants were advised to have their home UV treatment immediately before travelling to the University of Edinburgh’s Clinical Research Centre, where measurements were taken within 90 min of irradiation.

Skin colour was recorded on the volar forearm with a Minolta chromameter CM2500d (Osaka, Japan). The mean of triplicate measurements using the L.a.b system (Commission Internationale de l’Eclairage, CIE. http://members.eunet.at/cie/) was recorded. Pigmentation was scored using the L* measure which represents lightness on a scale of 0 to 100 where 0 is black and 100 is pure white [9].

Clinic BP was recorded with an automatic, calibrated sphygmomanometer (Microlife WatchBP Home, Widnau, Switzerland). After sitting at rest for 10 min, three serial BP measurements were taken with a 1-min pause between measurements. The mean of the second and third reading was recorded. 24-h ABP was measured from day 0–1 (before any irradiation) and day 14–15.

### Data handling and statistics

The change in 24-h ABP between day 0–1 and day 14–15 was calculated together with the change between days 1 and 15 clinic BP readings. Change in BP in the sham-irradiated treatment period was compared to change in BP in the active irradiated treatment period using paired *t*-tests with Minitab Statistical software (version 20.4). Change in L* score, vitamin D and nitrogen oxide levels were similarly handled.

## Results

**Fig. 1:**
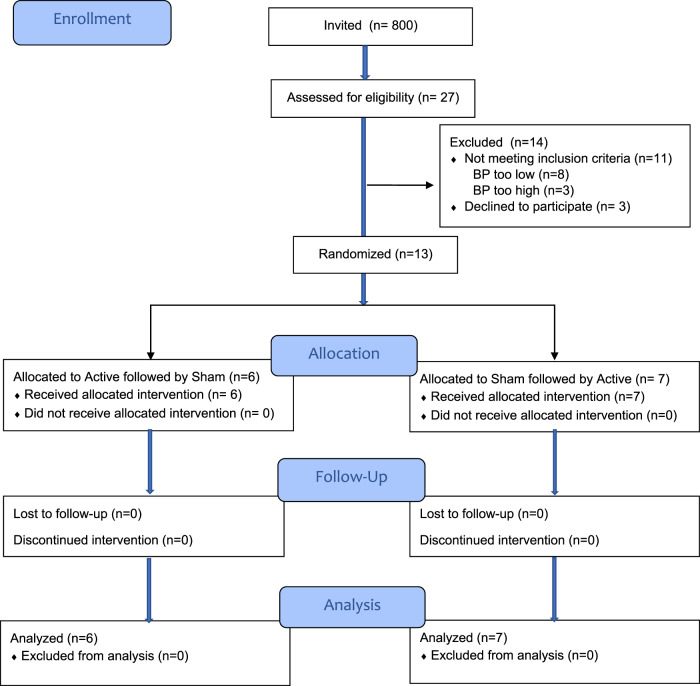
CONSORT flow chart of study enrollment. Enrollment and allocation information is summarised for invited patients.

**Fig. 2:**
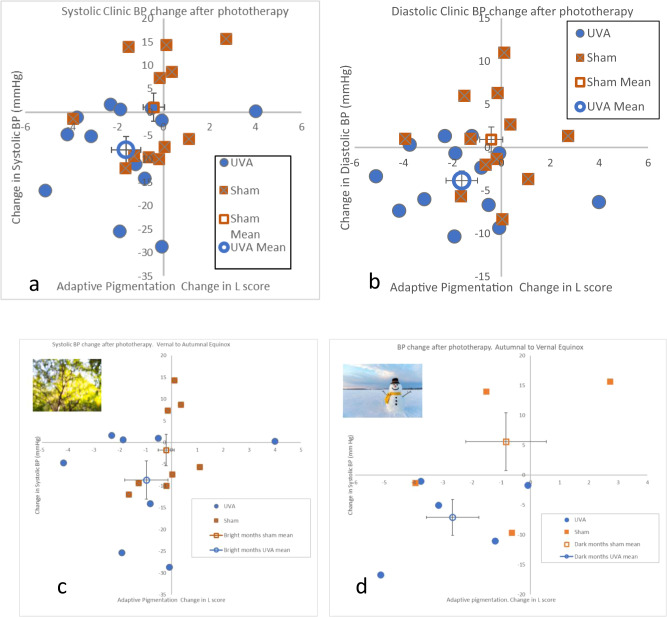
Change in clinic BP and skin pigmentation after 2 weeks phototherapy. Clinic BP measurement of change in systolic (**a**) and diastolic (**b**) BP and skin pigmentation after 2 weeks daily whole body active or sham irradiation with 5 J/cm^2^ UVA. Differences between sham and mean irradiation are significant (*p* = 0.034 for systolic and 0.029 for diastolic BP). **a**, **b** Measurements recorded over all seasons. **c** Systolic BP changes recorded in the lighter half of the year from Vernal to Autumnal Equinox. **d** Systolic BP changes recorded in the darker half of the year from Autumnal to Vernal Equinox. Error bars show sem.

We had significant problems recruiting patients. 800 patients registered as having mild hypertension were invited from eight general practices. Most of our local population in Edinburgh live in tenement flats, with no lift access and limited space to house a lamp. This limited the number of households to which heavy (40 kg) phototherapy lamps could be delivered, which severely hampered recruitment. Of those invited, 27 attended for screening, but 14 patients failed to meet the entry criteria. Three patients withdrew after the screening visit, one because they could not tolerate the 24-h ABP measurements and two for lack of time. Eight patients were found to be normotensive on clinic or 24-h ABP measurement, and three patients had BP higher than the inclusion criteria and were referred on for pharmacological BP management. Thirteen patients were enrolled, all of whom completed the study (Fig. 1). The mean age was 61.1 ± 8.6 years and BMI 27.7 ± 4.8. Fitzpatrick skin type was: six type 2, six type 3 and one type 4. The mean washout period between interventions was 20.0 ± 9.5 days. The study was underpowered to show anticipated changes in 24-h ambulatory BP, and no change was seen. We did, however, observe a fall in clinic BP following active phototherapy (−8.0 ± 2.9/−3.8 ± 1.1 mm Hg) with no change in the sham irradiation treatment period (1.1 ± 3.0 /0.9 ± 1.5 mm Hg) (Fig. 2a, b). The difference in BPs between active and sham irradiation was significant for both, systolic (*p* = 0.034) and diastolic BP (*p* = 0.029). Following active irradiation subjects developed subtle, but significant adaptive pigmentation represented by a fall in the L* score of −1.62 ± 0.64 (*p* = 0.023), while there was no significant change with sham irradiation (Fig. 2a). Those subjects treated with 2 weeks of UVA in the 6 months of the year with highest ambient sunlight (vernal equinox to autumnal equinox) tended to have a lower increase in adaptive pigmentation (change in L* −0.97 ± 0.84 Fig. 2c) than those in the darker half of the year (−2.67 ± 0.90 Fig. 2d). Serum 25(OH)D3 level changes over the 2-week intervention period for sham and active irradiation were −0.36 ± 5.05 and 11.61 ± 11.93 nmol/l (*p* = 0.0012). NO could not be measured directly, but plasma concentrations of the more stable oxidation products, nitrite and nitrate, showed no significant changes. The serum nitrite concentration change over sham and active irradiation periods was −0.01 ± 0.10 μM and 0.02 ± 0.08 μM. Serum nitrate concentration change over sham and active irradiation periods was 6.6 ± 14.8 μM and 0.7 ± 18.8 μM. However, patients were not on controlled diets, and dietary nitrate intake is the major determinant of circulating nitrogen oxide levels.

## Discussion

This is the first clinical trial to assess the possibility of using daily phototherapy to treat mild hypertension. We had unanticipated difficulty recruiting subjects, largely because of the problems of delivering lamps to subjects’ homes. Dermatology patients with an unsightly and highly symptomatic condition may be more accepting of the drawbacks of bulky phototherapy lamps than patients with asymptomatic and invisible hypertension. In any case, the practical challenges of home phototherapy mean that its use is largely restricted to geographically remote areas where access to hospital phototherapy units is difficult. Limited recruitment meant that the study was underpowered to detect our primary endpoint of reduced 24-h ambulatory BP. Nonetheless, we show a significant fall in clinic BP, our secondary endpoint. Clinic BP was recorded within 90 min of patients receiving their final UV irradiation at home and thus probably represents the acute fall in BP following a single UVA irradiation [7] rather than any chronic change, which would be seen with ABP. Our cohort of patients had high-normal or stage 1 hypertension [8]. Seasonal variation in BP is greater in those with more marked hypertension [10], and increased arterial stiffness [11]. Autoregulatory mechanisms may have prevented more sustained falls in BP in our cohort with this lowest level of hypertension.

Sustained reduction in BP is needed to reduce incident cardiovascular disease making it unlikely that UVA phototherapy as we have delivered it will reduce cardiovascular disease. The 5 J/cm^2^ UVA with which we irradiated subjects is equivalent to the amount in midday sunshine in December of 124 min in Edinburgh, or 79 min in London. The midday June equivalent would be 17 min and 16 min, although in summer there is also a UVB component to sunlight which was not emitted by our lamps.

NO is not stored biologically and has a half-life of seconds [12], but we have previously demonstrated stores of the more stable nitrogen oxides, nitrate (NO^−^_3_), nitrite (NO^−^_2_) and nitrosothiols (-SNO) in human skin at levels around ten times as high as those in the circulation [13]. Nitrate is a relatively stable end product of oxidation of NO, but another major source is dietary [14], such that a high nitrate diet can lower BP [15]. Nitrosothiols are photolabile, and nitrate and nitrite can also be photolysed by UV in the presence of thiols [16]. Arterial ‘photorelaxation’ was described by Furchgott over 60 years ago [17], and the action spectrum of NO release from rat aorta was shown to match that of nitrosothiols and nitrite, consistent with these being the vascular sources of NO-mediated ‘photorelaxation [18]. More recently we have demonstrated that UVA irradiation of ex vivo human skin slices causes a dose dependent release of NO [7]. Irradiation of human volunteers with 20 J/cm^2^ UVA leads to direct arterial vasodilatation, a fall in BP, and rise in heart rate and NO (indicated by measurement of its more stable oxidation product nitrite) [7]. The fall in blood pressure correlates with the UVA induced rise in circulating NO [19]. UVA was chosen for these experiments to demonstrate a vitamin D independent effect but this is not evidence that these wavelengths are the most effective at lowering BP. More recently, on irradiating isolated skin slices we have shown a peak of NO release in the UVB range [20], and in an observational study of environmental UV and BP, we have shown that the fall in BP is greater with UVB than UVA [5]. Vitamin D blood levels are a measure of the UVB fraction of sunlight. The strong inverse correlation between measured serum vitamin D levels and blood pressure is thus more indicative of an inverse correlation between environmental UVB and blood pressure than UVA. While UVA lamps are less likely to induce an erythema than UVB lamps, and thus safer to use at home, it may be that broadband UVB, or solar simulator lamps that match the sun’s spectrum may be more effective at lowering BP. Interestingly, vitiligo patients treated with long-term UVB phototherapy lamps have reduced cardiovascular and cerebrovascular mortality [21].

Interactions between dietary nitrate and environmental UV should be considered. Oral nitrate derived NO can reduce oxygen demand by enhancing mitochondrial efficiency [22] which is used by endurance athletes consuming naturally high nitrate foods, such as beetroot juice [23]. A combination of UVA irradiation and an oral nitrate load is more effective at improving elite cyclists performance than either intervention alone [24] suggesting an interaction between diet and UV exposure on NO mediated effects such as blood pressure regulation.

Subjects were given daily phototherapy for 2 weeks, followed by a washout period and then 2 weeks of the alternative intervention. This short intervention period was to reduce confounding by changes in background environmental UV during each treatment period of the study. Total daylight length varies by 3 h 47 min between the start and end of the 7-week period around the equinoxes in Edinburgh, reducing to no change in the 7-week period around the solstices. To minimise confounding by environmental UV changes over the course of the study we thus limited the overall study period to the shortest time in which we anticipated identifying an effect.

Significant seasonal variation in BP, myocardial infarction, stroke, and cardiovascular and all-cause mortality have been appreciated for the last 50 years with the effect size being similar to that produced by pharmacological agents [4]. UVB wavelengths of sunlight support the synthesis of vitamin D, which is essential to human health. While observational studies show an inverse relationship between serum vitamin D levels and BP [25], cardiovascular and cerebrovascular [26] disease, numerous well-conducted interventional studies and meta-analyses of these studies show no benefit from oral vitamin D supplements on these conditions [15–17, 27–29]. Mendelian randomisation studies have been less clear cut [30, 31] but the most recent synthesis suggests an association between the lowest vitamin D levels and coronary heart disease, but no effect above a threshold of 25 nmol/L [32]. This is in contrast to the strong, linear dose-dependent observational associations between measured Vitamin D and cardiovascular outcomes [33]. Thus, while low levels of vitamin D may account for some cardiovascular morbidity it is insufficient to account for all of the observed association. Our data suggest that Vitamin D is also a marker for sunlight exposure, which may act independently of vitamin D to lower BP and CVD. Although the F85/100 W UVA bulbs we used predominantly emit UVA wavelengths, enough radiation is produced within the vitamin D action spectrum to have produced a rise in 25(OH)D3 levels. This is not evidence for a causative role of vitamin D in lowering BP. During the active treatment period of the study, subjects’ skin darkened, but we are no more advocating the use of vitamin D supplements to treat hypertension, than application of fake tan. The strong inverse association between measured vitamin D levels and BP has unfortunately clouded the debate on sunlight and BP for many years and hidden the potential for the use of optimised UV itself in BP control. In our study, the skin darkening, and rise in measured vitamin D levels of subjects during the active, but not control intervention was confirmation of adherence to treatment.

Sun exposure is associated with a reduction in all-cause mortality [2]. BP reduction linearly predicts reduced cardiovascular events and deaths [34] and high BP is the leading global cause of DALYs. Malignant melanoma is the most prevalent lethal skin cancer, but the association between sunlight exposure and malignant melanoma is nonlinear, with intermittent burning sunlight in childhood predisposing to trunk and limb melanomas [35].

Our study failed to show a sustained hypotensive effect of a single moderate daily irradiation of UVA, although it confirmed that a transient fall in BP is produced. Summer reductions in blood pressure with the associated falls in cardiovascular deaths occur in the context of continuous exposure to full spectrum (UVA and UVB) sunlight. For those in high latitude countries, environmental UV is inadequate to produce a significant fall in BP in winter, and clothing worn to protect against the cold limits skin exposure to what UV there is. As our study has demonstrated, conventional fluorescent bulb technology is unlikely to be practicable as a source of artificial UV. Rapid advances in LED technology may produce a range of alternative more convenient and safer phototherapy sources. Further research on the action spectrum and pharmacokinetics of UV-related BP regulation may offer new therapeutic options for the treatment of high BP as we define more accurately the wavelength, dose, and length of exposure necessary to reproduce environmentally driven seasonal falls in BP.

### Summary

#### What is known about this topic

- Blood pressure and cardiovascular mortality are lower in summer than winter, independently of vitamin D and only partly due to temperature change.
- UVA irradiation releases the vasodilator NO from stores in the skin to the systemic circulation.

#### What this study adds

- A low dose of daily whole body UVA for 2 weeks does not reduce 24 h ambulatory BP, but does reduce clinic BP measured within 90 min of irradiation.
- Higher fluences of UVA, or exposure to UVB wavelengths, may account for summertime fall in BP.

## Data Availability

Additional data are available from the corresponding author on reasonable request.
